# Focal radiation necrosis of the brain in patients with melanoma brain metastases treated with pembrolizumab

**DOI:** 10.1002/cam4.1726

**Published:** 2018-08-21

**Authors:** Stephanie Du Four, Yanina Janssen, Alex Michotte, Anne‐Marie Van Binst, Robbe Van den Begin, Johnny Duerinck, Bart Neyns

**Affiliations:** ^1^ Department of Neurosurgery Universitair Ziekenhuis Brussel Brussels Belgium; ^2^ Department of Medical Oncology Universitair Ziekenhuis Brussel Brussels Belgium; ^3^ Department of Neurology and Neuro‐Pathology Universitair Ziekenhuis Brussel Brussels Belgium; ^4^ Department of Radiology Universitair Ziekenhuis Brussel Brussels Belgium; ^5^ Department of Radiotherapy Universitair Ziekenhuis Brussel Brussels Belgium

**Keywords:** immunotherapy, melanoma brain metastases, pembrolizumab, radiation necrosis

## Abstract

**Introduction:**

Up to 60% of patients with metastatic melanoma develop melanoma brain metastasis (MBM) during the course of their disease. Surgery, radiosurgery (SRS), stereotactic radiotherapy (SRT), and whole‐brain radiation therapy (WBRT) or combinations of these are commonly used local treatment modalities. Inhibitory monoclonal antibodies against the CTLA‐4 and PD‐1 immune checkpoint receptors significantly improved the survival of metastatic melanoma patients, including patients with MBM. This prolonged survival, and potentially also the immunostimulatory mechanisms, may expose patients to a higher risk for long‐term complications such as focal postradiation necrosis of the brain (RNB).

**Methods:**

We analyzed the incidence of pseudotumoral RNB in a single institution cohort of 142 melanoma patients that were prospectively followed after starting treatment with pembrolizumab in an expanded access program.

**Results:**

Of the 142 patients, 43 (30.7%) patients had MBM at initiation pembrolizumab. Of these, 31 (72.1%) were treated with SRS, 8 (18.6%) with WBRT while 4 (9.3%) had no prior local therapy. Of patients treated with RT, 28 (71.1%) received RT before the initiation of pembrolizumab. 5 (12.8%) patients developed a new symptomatic pseudotumoral lesion at a median time of 11.15 months (range 8‐46) after the RT. In all patients, the diagnosis of RNB was radiologically confirmed. The RNB was treated with corticosteroids in two patients, bevacizumab in two patients, and surgery in three symptomatic patients. The diagnosis was histologically confirmed in the patients treated with surgery.

**Conclusion:**

Melanoma patients with MBM treated with radiosurgery and showing a beneficial response to pembrolizumab are at risk for late RNB. In case of suspected isolated progression at the site of a previously irradiated MBM, the diagnosis of RNB should be considered.

## 1 INTRODUCTION

Melanoma brain metastases (MBM) are a common devastating consequence of metastatic melanoma. About 7% of metastatic melanoma patients have MBM at first diagnosis, and about 40%‐60% of metastatic melanoma patients develop MBM during the course of their disease. The median survival of these patients was about 4‐6 months prior to recent development of targeted therapy.^1^, ^2^

Before the availability of effective systemic treatment options, local treatment of brain metastases was standard of care. Surgical resection and whole‐brain radiation therapy (WBRT) alone have limited efficiency in obtaining local control of MBM.^3^ Radiosurgery (SRS) and stereotactic radiotherapy (SRT) can be effective local treatment options in selected patients with a limited number of small (<3 cm) MBM and may improve survival.^4^, ^5^, ^6^ The addition of WBRT to SRS/SRT has led to a survival benefit in patients with a good performance status and brain metastases from non‐small‐cell lung carcinoma.^7^ However, in melanoma it has not been shown to further increase overall survival. Although the neurocognitive toxicity is lower than in WBRT, SRS/SRT can lead to radiation necrosis of the brain (RNB), being local radiation‐induced brain damage with demyelination, edema, and eventually necrosis of normal tissue. The incidence of melanoma RNB has been about 5%‐6.6% in prospective studies, usually has a delay of 6‐30 months after radiotherapy.^4^, ^5^, ^8^

About 50% of melanoma patients have a BRAF mutation. In these patients, the combination treatment with BRAF and MEK inhibitors is a standard of care.^9^, ^10^, ^11^ In prospective and retrospective trials, it has been shown that dabrafenib and vemurafenib have activity against previously treated and untreated MBM.^12^, ^13^, ^14^ Recently, a phase 2 randomized clinical trial investigating the effect of the combination dabrafenib/trametinib in patients with MBM showed an intracranial response rate of 58%. The asymptomatic patients who were previously treated with local therapy had the best progression‐free survival (7.2 months). These data support the use of combination therapy in patients with BRAF‐mutated MBM.^15^

For metastatic melanoma patients without BRAF mutation, therapy with immune checkpoint inhibitors has become first choice. Ipilimumab, an anti‐CTLA‐4 monoclonal antibody, was the first to show improved survival in these patients.^16^, ^17^ In a phase 2 clinical trial, it has been shown that ipilimumab has activity in patients with stable, asymptomatic MBM who are independent of corticosteroid treatment.^18^ The anti‐PD‐1 monoclonal antibodies, nivolumab and pembrolizumab, have also led to durable responses and significantly longer overall survival. Moreover, the combination of anti‐CTLA‐4 with anti‐PD‐1 led to a significantly longer progression‐free survival and higher objective response rate as compared to ipilimumab alone.^19^, ^20^, ^21^, ^22^ The preliminary results of a randomized phase 2 clinical trial investigating the effect of the combination of nivolumab with ipilimumab and nivolumab alone in patients with MBM (asymptomatic, without local radiotherapy) showed an intracranial response of, respectively, 42% and 20% and a PFS of, respectively, 4.8 and 2.7 months. In previously treated symptomatic patients, the intracranial response was 6% with a PFS of 2.5 months. Another nonrandomized clinical trial showed similar results with the combination of nivolumab and ipilimumab in patients with asymptomatic previously treated MBM, that is, an intracranial response of 55% (PFS not reached). These results show that the combination treatment has high activity in asymptomatic patients with MBM.^23^, ^24^

We previously reported several cases of RNB in patients successfully treated with ipilimumab.^25^, ^26^ Given the even further improved survival of metastatic melanoma patients with available anti‐PD‐1 treatments, including patients with MBM, more patients may be at risk for long‐term complications of RT such as focal RNB.

Recently, Colaco et al retrospectively examined the incidence of RNB after gamma knife treatment for brain metastases. After uni‐ and multivariate analyses, they found an increased rate of RNB in patients who were treated with systemic immunotherapy alone. Moreover, the patients who developed RNB had a significantly longer OS as compared to the total population. With the improving use of immunotherapy and the increased overall survival of patients with brain metastases, they foresee an increased rate of RNB in the future after radiosurgical treatment.^27^

Here, we report the incidence and case study of RNB in a prospectively followed cohort of metastatic melanoma patients treated with the anti‐PD‐1 monoclonal antibody pembrolizumab at a single institution.

## 2 MATERIAL AND METHODS

We analyzed the incidence of delayed pseudotumoral RNB in a cohort of 142 melanoma patients treated with pembrolizumab (2 mg/kg administered intravenously every 3 weeks) in an extended access program between September 2014 and September 2016. The expanded access program (EAP) was approved by our institutional independent ethics committee, and all patients gave written informed consent for participation in a simultaneous academic noninterventional clinical trial, that prospectively collected baseline and outcome data from patients treated with pembrolizumab in the EAP. This clinical trial was also approved by the institutional independent medical ethics committee of the UZ Brussel.

In patients where there was a suspicion of radiation necrosis, the diagnosis was initially made on a cerebral MRI. Conventional MRI does not provide sufficient information to differentiate radiation effects from tumor recurrence. We used MR perfusion (DSC perfusion) and MR spectroscopy (MRS) in differentiating recurrent tumor from radionecrosis, based on parameters as rCBV and ratios of choline/N‐acetyl aspartate (Cho/NAA). Areas of enhancement do not show increased rCBV in radiation necrosis and can be helpful in distinguishing them from recurrence (a maximum lower cutoff for rCBV of 1.5 for radionecrosis and usually over 2.2 for tumor recurrence). MRS typically shows low choline, creatine, and NAA in radionecrosis. Lipid peaks can also be seen. In tumor recurrence, high choline peaks are seen and low NAA (Cho/NAA over 2).

## 3 RESULTS

Of the 142 patients that initiated pembrolizumab between September 2014 and September 2016, 43 (30.7%) had MBM at the start of treatment. The baseline patient population characteristics are summarized in Table 1. The mean age was 53 years (range 33‐84 years) with a predominance of female patients (66.7%; F/M: 29/14). The majority of patients (67.4%) were previously treated with ipilimumab. Thirty‐nine (90.7%) of the 43 patients were treated with RT for their MBM. The RT was performed before the initiation of pembrolizumab in 28 (71.7%) patients and after the pembrolizumab in 11 patients (28.3%). The median time between RT and pembrolizumab treatment was, respectively, 5.8 months (range 0.4‐25.8) and 1.2 months (range 0.1‐46.3).

**Table 1:** Population characteristics of patients with brain metastases (n = 43)

Median age (years)	50 (33‐84)
Sex (F/M)	29/14
Primary site melanoma	
Skin	33 (76.7%)
Unknown	10 (23.3%)
Best tumor response on pembrolizumab	
CR	3 (7.0%)
PR	4 (9.3%)
SD	10 (23.3%)
PD	25 (58.1%)
Not evaluable	1 (2.3%)
Previous treatment with ipilimumab	
No	14 (32.6%)
Yes	29 (67.4%)
Radiation therapy for MBM	
None	4 (9.3%)
SRS	31 (72.1%)
WBRT	8 (18.6%)
Radiation therapy	
Before pembrolizumab	28 (71.7%)
During pembrolizumab	11 (28.3%)

Most patients (31 pts, 72.1%) were treated with SRS as compared to WBRT (8 pts, 28.3%). Four (9.3%) patients were treated twice with SRS for the occurrence of new MBM. Five (12.8%) of the 39 patients treated with radiotherapy developed RNB. The majority of patients who developed RNB were treated with RT after the start of pembrolizumab.

The best overall tumor response to treatment with pembrolizumab of patients with MBM, according to the immune‐related response criteria (irRC), was a complete response (CR) in 3 pts (7.1%), partial response (PR) in 4 pts (9.5%), stable disease (SD) in 9 pts (21%), and progressive disease in 25 pts (58.1%) (Table 1).

**Figure 1:**
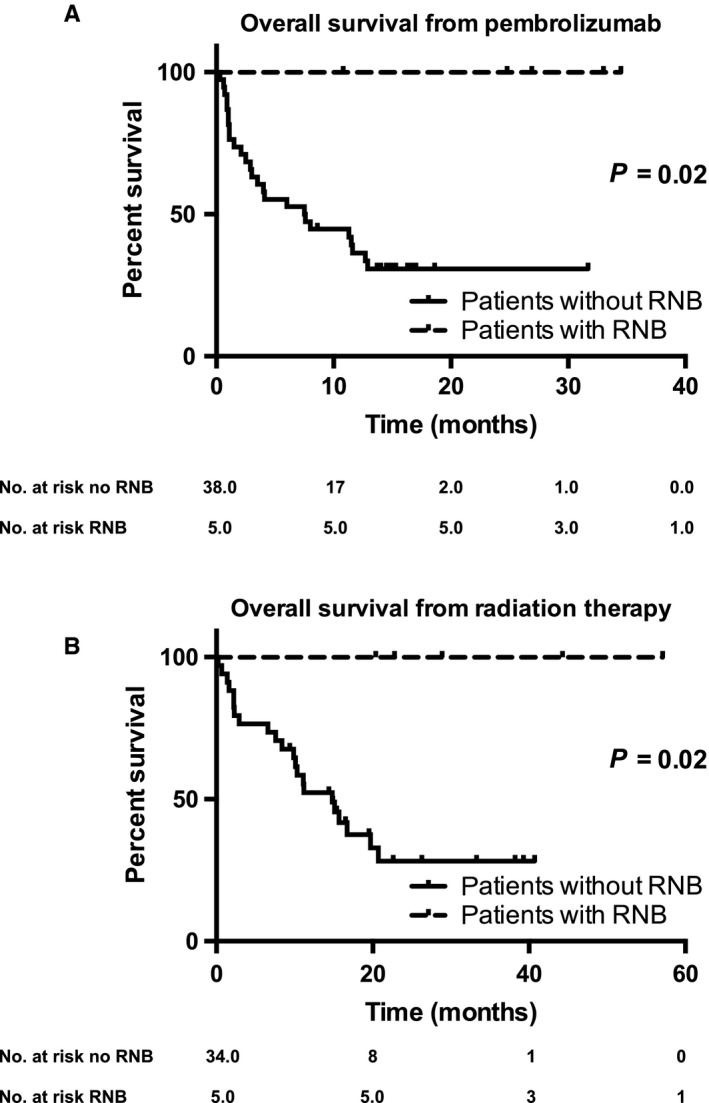
Kaplan‐Meier curves of patients with MBM treated with pembrolizumab. Comparison of Kaplan‐Meier curves between the patients without MBM treated with pembrolizumab and patients who developed RNB (A) from the start of pembrolizumab treatment; (B) from the start of radiation therapy

At the time of analysis, after a median follow‐up of 50 months (range 33‐84), 26 (61.9%) patients had died. The median overall survival from the start of pembrolizumab was 11.3 months (Figure 1A), while the median OS from RT is 15.7 months (Figure 1C). The median time from RT to the diagnosis RNB is 11.15 months (range 8‐46), and the median time from the start of pembrolizumab to RNB is 7.1 months (range 0‐20) (Table 2). All patients who developed late RNB are still alive at the time of analysis. Their best OR was CR (1 pt), PR (1 pt) and SD (3 pts). These patients showed a significantly longer overall survival as compared to the patients who did not develop RNB (*P* = 0.02; Figure 1B,D).

**Table 2:** Overview of patients who developed radiation necrosis

Patients	Age (years)	MBM (no)	Time between RT and RNB (months)	Time between pembro and RNB (months)	Time between RT and pembro (months)	Treatment
1	52	4	9.2	9.2	2.0	Surgery bevacizumab
2	63	4	11.1	1.4	9.8	Surgery
3	43	1	8.2	20.8	−12.7	Bevacizumab
4	53	4	12.4	14.4	−2.0	Steroids
5	48	2	46.2	0	46.3	Surgery bevacizumab

### 3.1 Case 1

A 52‐year‐old man was first diagnosed with melanoma in 2008 (Clark level IV, Breslow 0.61). In February 2015, he was diagnosed with multiple MBM (one frontal left, two frontal right, one occipital right), metastases of the cervical spine (C2, C5, C6), and a lymph node metastasis in the neck. A biopsy of the lymph node metastasis showed the absence of a BRAF mutation. A treatment with ipilimumab was initiated (3 mg/kg every 4 for weeks. The MBM were each treated with SRT (20 Gy in one fraction). After three cycles of ipilimumab, the patient had a progressive intracranial and extracranial disease. A treatment with pembrolizumab was initiated at a dose of 2 mg/kg.

**Figure 2:**
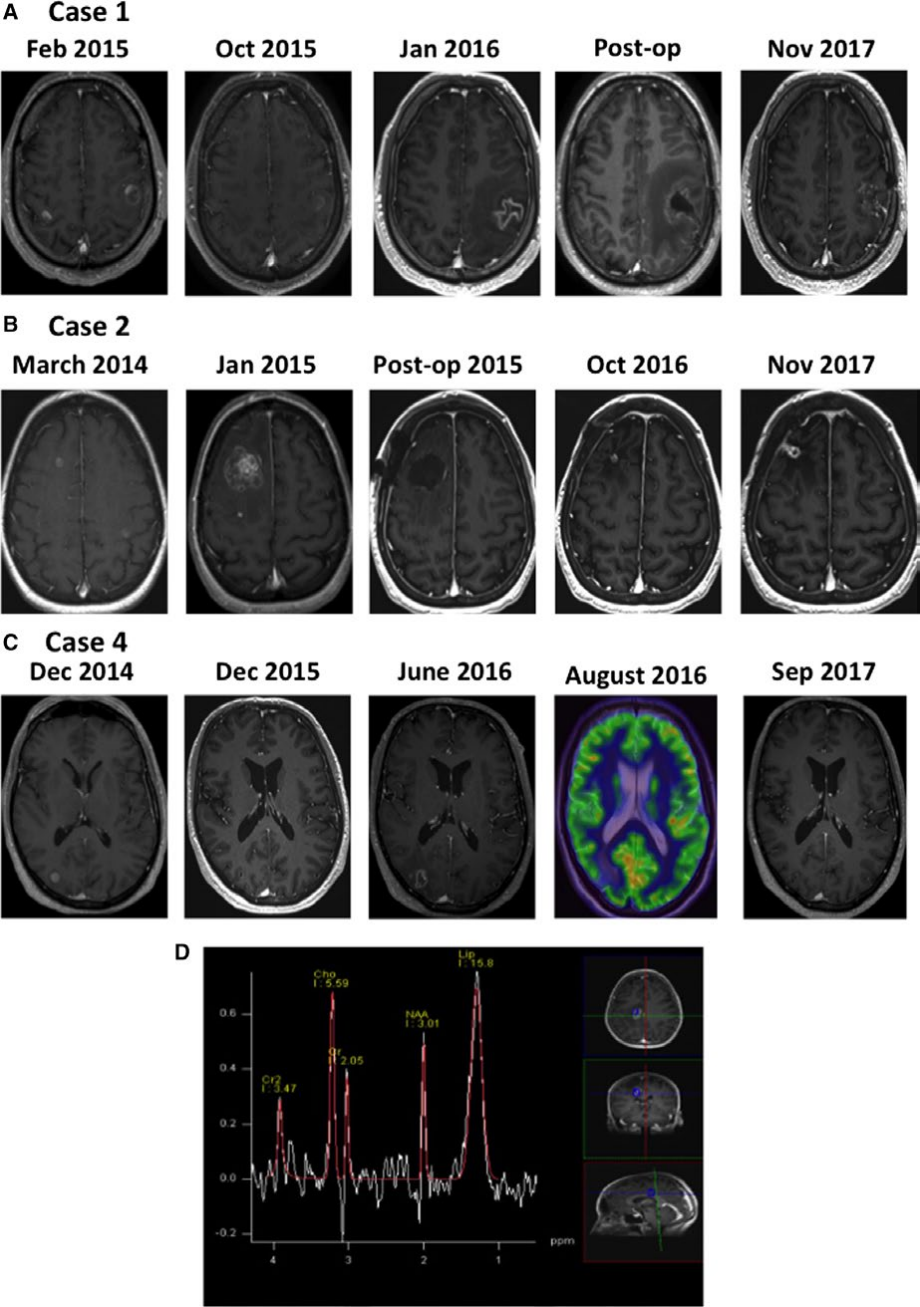
Evolution of brain lesion from before RT, after RT and development of RNB until current status of: A, case 1; B, case 2; and C, case 4. D, MR spectroscopy of case 3 showing a spectrum typical for RNB

A cerebral MRI in December 2015 showed an increase in volume of the left frontal lesion (Figure 2A). As the patient had a complete remission of the extracranial disease and the other MBM remained stable, there was a suspicion of RNB. Further investigations with an MR spectroscopy were inconclusive in differentiating between RNB and disease progression.

In January 2016, the patient developed complex partial epileptic seizures. A treatment with corticosteroids was started. Due to a worsening neurological symptoms and increasing size of the lesion, a surgical resection was indicated. The histology confirmed the presence of RNB. In July 2016, the patient developed a paresis of the right hand due to increasing edema surrounding the resection cavity. The patient had a good clinical response after initiation of corticosteroids. However, after tapering he had a recurrence of neurological symptoms in October 2016. A treatment with bevacizumab at 5 mg/kg was initiated during 4 cycles. The corticosteroids could be stopped with a neurological stabilization; however, 5 months later he had an increased neurological deficit. Thereupon bevacizumab treatment was resumed with symptomatic improvement.

Until the last follow‐up in December 2017, the patient remained in complete intracranial (Figure 2A) and extracranial remission after 14 cycles of pembrolizumab.

### 3.2 Case 2

In March 2012, a 63‐year‐old woman was diagnosed with a melanoma on the right arm (Clark IV, Breslow 2.5 mm). In August 2013, a treatment with DTIC/carboplatinum was initiated for the diagnosis of a metastatic melanoma (stVIM1c). After two cycles, the patient had a progressive disease with the diagnosis of two MBM. In November 2014, these were treated with SRT (20 Gy, 1 fraction). At that time, treatment with ipilimumab (3 mg/kg) was initiated. In March 2014, two new asymptomatic MBM were diagnosed and treated with SRT (20 Gy, 1 fraction). For progressive disease, a systemic treatment with fotemustine was initiated in June 2014. After for a total of three cycles, a treatment with pembrolizumab (2 mg/kg) was started. Over several months, a right frontal MBM increased in size with an increase in perilesional edema (Figure 2B). In January 2015, there was a further increase in size and edema and a surgical resection was indicated (Figure 2B). The histological analysis confirmed the presence of RNB (Figure 4). After the surgery, the corticosteroids could be stopped. Currently, the patient is still treated with pembrolizumab and has no active intracranial or extracranial disease.

**Figure 3:**
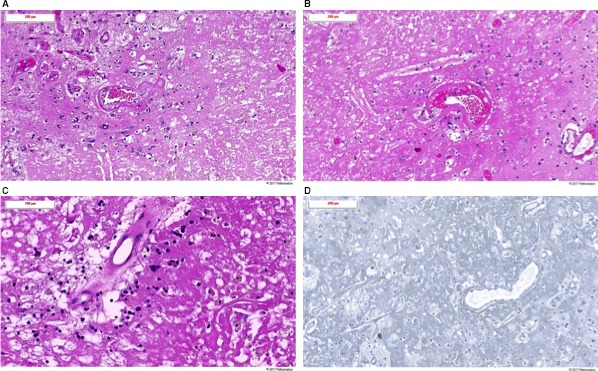
Histological images of radiation necrosis after surgical resection (case 5). Histological images of hematoxylin and eosin stain: A, Ill‐defined area of amorphous necrosis without any residual melanoma cells. Some vessels show histological signs of therapy‐induced fibrinoid necrosis. Presence of reactive astrocytes (gliosis) in the neighboring brain tissue associated with scattered histiocytic cells and slight lymphocytic inflammatory reaction. B, Ill‐defined area of amorphous necrosis. Radiotherapy‐induced fibrinoid necrosis of some vessels. C, Amorphous necrosis, radiotherapy‐induced vascular changes, scattered hemosiderin‐laden histiocytes and lymphocytes. D, Histological images of HMB45 immunostaining. No residual melanoma cells can be identified by the HMB45 immunostaining

**Figure 4:**
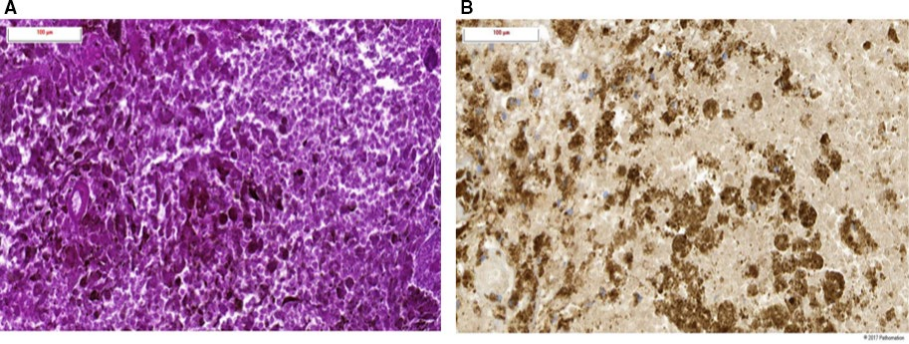
Histological images of radiation necrosis after surgical resection (case 2). A, Histological images of hematoxylin and eosin stain. Area of diffuse necrosis with residual tumoral cells containing neuromelanin. B, Histological images of Melan‐A immunostaining showing residual neuromelanin containing tumoral cells

### 3.3 Case 3

A 43‐year‐old woman was diagnosed with a BRAF wild‐type metastatic melanoma in 2014. After surgical resection of a single lymph node metastasis, the patient was recruited for the DC‐MEL study examining the effect of an autologous dendritic cell vaccination in patients with stage III/IV melanoma without measurable disease (EudraCT number: 2011‐001410‐33). In June 2014, a treatment with ipilimumab (3 mg/kg) was initiated for progressive disease. In September 2015, she developed liver and brain metastases. A treatment with pembrolizumab at 2 mg/kg was immediately initiated en the solitary MBM was treated with SRT in October 2015 (20 Gy). In April 2016, a complete extracranial remission was obtained. In June 2016, the patient experienced complex partial epileptic seizures followed by a paresis of the left foot. The cerebral MRI showed an increased volume of the lesion with important perilesional edema, which was suggestive for RNB. An MRI spectroscopy was performed that confirmed the diagnosis of RNB. After a treatment with corticosteroids, the patient had a good clinical response; however, in August 2016 she had increasing epileptic seizures. A treatment with bevacizumab (7.5 mg/kg, every 3 weeks, 4 cycles) was initiated with a good clinical and radiological response. Until latest follow‐up, the patient has no clinical or radiological signs of disease recurrence.

### 3.4 Case 4

A 53‐year‐old man was randomized in the DC‐MEL trial (EudraCT number: 2011‐001410‐33) in May 2014 after the resection and radiation of lymph node metastases of a BRAF wild‐type melanoma. In December 2014, he developed recurrent lymph node metastases and a left occipital MBM. A treatment with Trimix DC vaccination and ipilimumab (3 mg/kg) was initiated. After four cycles, he was progressive and a treatment with pembrolizumab (2 mg/kg) was started. Due to the development of an autoimmune colitis and orchitis, the treatment with pembrolizumab had to be stopped after 4 cycles. In June 2015, he developed a right parietal MBM that was treated with SRT (1 × 20 Gy). One month later, the right parietal lesion disappeared; however, there were two new cerebral lesions. These were also treated with SRT (1 × 20 Gy). One month later, a treatment with temozolomide (autoimmune side effects) was initiated for a progressive extracranial disease. In June 2016, there was an increase in volume of the right parietal brain lesion. The cerebral MRI was suggestive for RNB. The FDG‐PET CT showed the presence of hypometabolic lesion, which strongly suggests RNB (Figure 2C). The patient remained asymptomatic, and no specific treatment was necessary. The patient remains in complete remission.

### 3.5 Case 5

In July 2011, a 48‐year‐old woman had a bilateral mastectomy with axillary lymph node resection for metastases of a BRAFV600E‐positive melanoma followed by a treatment with vemurafenib. After 11 months of treatment, the patient had progressive disease. A treatment with ipilimumab (10 mg/kg) and DC vaccination was initiated (TriMixDC‐MEL plus ipilimumab).^28^ In November 2012, the patient developed a MBM frontal right that was treated with SRT (1 × 20 Gy). After 18 months of maintenance therapy with ipilimumab, the treatment a rechallenge with vemurafenib/trametinib was initiated for progressive disease. In October 2016, pembrolizumab was initiated for a progressive disease. The cerebral MRI at that time showed an increase in volume of the right frontal metastasis with significant perilesional edema. The appearance was suggestive for RNB. In absence of neurological symptoms, no specific treatment was initiated. A stable intra‐ and extracranial disease was obtained. In June 2017, the right frontal lesion further increased. The 18‐FET‐PET imaging showed a hypometabolic activity frontal right, which confirmed the presence of RNB. In July 2017, the patient developed headaches and a transient neurological deficit. A treatment with corticosteroids was initiated with an improvement of the neurological condition. Because a tapering of corticosteroids was impossible, the treatment with pembrolizumab was interrupted and a treatment with dabrafenib/trametinib was initiated.

Despite the treatment with corticosteroids, there was a further neurological deterioration in September 2017. Hence, a neurosurgical resection was performed in October 2017. The histological analysis confirmed the presence of radiation necrosis without signs of residual disease (Figure 3). Postoperatively, the corticosteroids could be tapered and stopped; however, the patient developed increasing headaches and a left hemiparesis. The cerebral MRI showed an increased contrast enhancement in the resection cavity and increasing perilesional edema. As there was only a minor improvement after reinitiating corticosteroid therapy, a treatment with bevacizumab was initiated in November 2017. Under this treatment, the patient had a remarkable neurological improvement with the recuperation of the left hemiparesis. Until latest follow‐up, the patient had a systemic and radiological stable disease.

## 4 DISCUSSION

In our prospective single institution series on the incidence of RNB in metastatic melanoma patients treated with pembrolizumab and previously treated with radiotherapy, the incidence of RNB was 12.8% (after a median follow‐up of 50 months). In all 5 RNB cases, the patients were alive at the latest follow‐up (with a median of 26.9 months [range 10.8‐34.5] after the initiation of pembrolizumab). Our series reflects the first real‐life daily practice in the treatment of patients with MBM.

Differential diagnosis of RNB with in‐field melanoma recurrence can be challenging. The diagnostic criteria of RNB are not well defined yet. However, it has been shown that MRI perfusion and spectroscopy are effective in diagnosing RNB.^29^, ^30^ In our cases, the diagnosis of RNB was based on diagnostic MRI mostly combined with MRI perfusion and spectroscopy. In addition, in all patients there also was an initial regression followed by a regrowth of the lesion. Recently, new imaging techniques have been described for the diagnosis of RNB. However, until now, none have been validated.^31^ Additional imaging with PET using 18F‐FDG and 18F‐FET has shown to help differentiate RNB from disease progression.^32^, ^33^ If possible, the diagnosis of RNB should be confirmed by histology, especially in patients with progressive pseudotumoral lesions and uncontrolled clinical symptoms.

Historically, RNB is treated with corticosteroids, often in high dosage and during a long period, leading to a high incidence of complications. Moreover, immunotherapy is often not compatible with a long‐during treatment. Over the past few years, the anti‐VEGF monoclonal antibody bevacizumab has been proposed as treatment for RNB. The rationale for treatment with bevacizumab lays in the underlying pathophysiological process.^34^ There are limited data concerning the use of bevacizumab in RNB. Recently, a meta‐analysis showed that bevacizumab treatment in patients with RNB leads to a reduction or stabilization of corticosteroid treatment in 97% of patients, and a clinical improvement of the neurological symptoms in 91.2% of patients. There were only 2.4% of grade three adverse events.^35^ A single‐arm prospective trial also showed a rapid decrease in perilesional edema after initiation of bevacizumab on MRI imaging.^36^ Moreover, Glitza et al^37^ reported that bevacizumab was safe in the treatment of RNB in patients with melanoma brain metastases treated with immunotherapy.

Several reports have shown an increased median overall survival in patients with MBM treated with RT and ipilimumab. Although it concerns retrospective trials and patient populations with a good performance score, these results show that immunotherapy leads to promising results even in patients with MBM.^38^, ^39^, ^40^, ^41^

Recently, it has been shown in a retrospective trial that nivolumab treatment in patients with MBM leads to a median overall survival of 9.9 months. Similar to the results with ipilimumab, patients who are corticosteroid independent and with asymptomatic MBM had a better progression‐free survival and median overall survival.^42^ A retrospective analysis of RT for MBM in a patient population treated with nivolumab has shown a good local and distant control and a median overall survival of about 12 months. Similar to the studies with ipilimumab, the performance status was predictive of a good overall survival.^43^

Although the majority of these studies are retrospective, the results are promising for the treatment and prognosis of patients with MBM. Although the majority of these retrospective trials did not investigate the incidence of RNB, Kiess et al^41^ report that the histologic analysis of five patients who had surgery for a suspicion of progression showed the presence of necrosis and lymphocytic inflammation. In the retrospective study with nivolumab treatment, the authors conclude that the follow‐up may not be long enough to capture the cases of RNB.^43^ Recently, a retrospective analysis of patients with brain metastasis of NSCLC, melanoma and RCC treated with immunotherapy showed an association between immunotherapy and the development of symptomatic RNB, especially in melanoma.^44^

In our patient population, the median overall survival was 11.3 months from the start of pembrolizumab. This is similar to the results obtained by Ahmed et al^43^ with nivolumab treatment and RT. However, we report the development of RNB in five of 39 patients. This is probably due to the fact that our population was first treated with RT before the initiation of the anti‐PD‐1 treatment. The patients who developed RNB also had a significantly longer overall survival, which makes them more prone for the development of RNB. Skrepnik et al found similar results in a population of 25 patients treated with ipilimumab and RT. They report a radiographic RNB in nine patients (20.7%), which was symptomatic in 5% of patients. The patients who developed RNB also had a significantly longer median OS. Moreover, they also suggest an optimal window of co‐administration of SRS and ipilimumab of <30 days.^45^

Recently, Silva et al investigated the long‐term neurotoxicity of RT in patients with MBM. In a patient population treated with anti‐PD‐1 and RT with a survival longer than 1 year after initiation of anti‐PD‐1 and RT, they found that 18% of patients (21 of 118) developed RNB as defined by radiology or histology.^46^ In our case series, we found a similar percentage of 16.1% RNB, if we take in count that all patients who developed RNB were treated with SRS (five out of 31 patients treated with SRS). Although it concerns a selected population of long‐term survivors, these results confirm that patients treated with immunotherapy with good response are at risk for the development of RNB.

There is not much data available on the activity and RNB risk in patients treated with anti‐PD‐1, or the combination of anti‐PD‐1 and anti‐CTLA‐4 mAb, and RT. Further prospective studies are necessary to investigate these outstanding questions.

## 5 CONCLUSION

In conclusion, patients with MBM treated with radiation treatment and beneficial response to immunotherapy are at risk for the development of RNB. In the case of recurrent disease during follow‐up, the diagnosis of RNB should be included in the differential diagnosis, especially in cases where this progression occurs as an isolated lesion in a previously irradiated location of the brain.

## CONFLICT OF INTEREST

There are no conflict of interests concerning this study. Possible conflict of interests for BN: Honoraria—Bristol‐Myers Squibb; Merck Sharp & Dohme; Novartis; Roche. Consulting or Advisory Role—Bristol‐Myers Squibb; Merck Sharp & Dohme; Novartis; Roche. Speakers’ Bureau—Novartis. Research Funding—Merck KGaA (Inst); Novartis (Inst); Pfizer (Inst). Travel, Accommodations, Expenses—Amgen; Bristol‐Myers Squibb; Merck Sharp & Dohme; Novartis; Roche.
